# Supplementary material for: The Virtual Navigation Toolbox: Providing tools for virtual navigation experiments
Source: PLoS One. 2023 Nov 9;18(11):e0293536. doi: 10.1371/journal.pone.0293536 (PMC10635524; doi:10.1371/journal.pone.0293536)
Supplement: Attachment — Submitted filename: Response to Reviewers.pdf [file pone.0293536.s001.pdf]

PONE-D-23-13307
The Virtual Navigation Toolbox: Providing tools for virtual navigation experiments
Response to Reviewers

21st August 2023

Summary Response

We are happy that all reviewers see the potential of the VNT to aid in the development of VR-driven spatial cognition experiments and we thank the reviewers for their detailed and constructive feedback to improve our manuscript. In collating the comments from the different reviews, we have taken as the central point of all reviews the observation that the initially submitted manuscript sat in an awkward intermediate spot in between a full introduction of our toolbox and a traditional empirical study. We agree with this assessment and have restructured the entire manuscript to fully conform to the format of a method showcase. This necessarily required several larger changes to the text, however, we feel these changes bring the manuscript more in line with our intention to present a comprehensive introduction to our toolbox. We will go over the different changes made to the manuscript below, as we respond to the reviewers' individual comments.

1 Review 1

> *I think that the tool proposed by the authors could be, in the future, very interesting in the context of spatial cognition. I can only agree with the principle of cooperative sharing of code and tools that could benefit the entire field. However, the presentation of the tool and the article in general seem to me a bit shaky as they are, and could benefit, in my opinion, from modifications.*

1.1 General structure

> *... the article follows the traditional structure "introduction / materials and methods / results / discussion", which seeks to resemble the format of empirical studies, but lacks the problematizations, objectives, and hypotheses but also the theoretical content to be one. I would advise to abandon this structure and do something more straightforward and more precise on the presentation of the tool according to the users' needs.*
>
> *I find that the article does not really support / and or discuss the use of the tool itself. I personally imagine much more a presentation with sections driven like "Why virtual reality is of interest in the field of spatial cognition, examples" ⟶ "How VR work in these cases and what needs for research, examples" ⟶ "VNT proposal and presentation" ⟶ "VNT testing through the showcase study" ⟶ "Discussion on the use of VNT in the showcase study and succinct reporting of the study's results" ⟶ "Global discussion on VNT". The article already looks in many ways like this but I'll take it all the way.*

Response

We agree with this assessment and have reworked the manuscript accordingly. Specifically, we have

- updated the introduction to give a better overview of VR research and a better explanation for the niche filled by our toolbox (section "VR: the "new" frontier in spatial cognition", ll. 10-29),
- restructured the main body of the manuscript to be more clearly centred on introducing the components of our toolbox and how they can be used,
- made it more explicit that the showcase study is to be seen in this context, rather than as a full empiricial study (ll. 118-121, reduced detail in section "Results of the showcase study" (ll. 538-566), and reduced detail in section "Evolving the pointing task: Embedded 3D marker placement" (ll. 630-646)),
- updated the discussion/outlook part to be more comprehensive overall (ll. 576-684).

We will address several of the changes made in more detail when responding to further reviewer comments relating to these issues.

1.2 Presentation and placement of the showcase study

> *In addition, the place of the showcase study in the article is a bit strange at the moment: I think it should exist only to make you think about the global tool presented here: the needs, what has been allowed, how it would have been otherwise without VNT, how did VNT benefit from the study etc. In this context, the showcase study should not be, in my opinion, a scientific empirical study in the "true" sense of the word (e.g. using null hypothesis testing via statistical inferences) and should not be presented as such (I'm not sure about making statistical analyses), but a demo accompanying the presentation of the tool (with, if wanted, descriptive statistics rather than inferential).*
>
> *Finally, and like many epistemologists and statisticians, I am opposed to the use of the terms "significant / non-significant" to talk about the results of the showcase study, especially considering its deep exploratory nature [Amrhein et al., 2019, Colquhoun, 2017].*

Response

We updated the manuscript throughout, to make clear that the showcase study is meant to be seen merely as a proof-of-concept for the presented toolbox (ll. 118-121). Furthermore, we agree that the exploratory nature and small sample size of our showcase study preclude any meaningful interpretation and have therefore removed all inferential statistics (ll. 538-566).

1.3 Code Re-usability

> *... it remains debatable for an experienced programmer that starting from scratch is sometimes more comfortable and faster than adopting and appropriating an existing code, especially in the absence of a precise and complete documentation. I think here that the authors should insist more on the proposed global "package" which allows to provide researchers with a whole set of tools which they would not necessarily have thought of but which can prove to be fundamental in the field, for example the dynamic recording of the positions and rotations of the participants (euler angles). Indeed, proposing a whole set of functions already established allows researchers to have a much wider range of functions at their disposal than what would have been built from scratch for the needs of a study, and this range could even push the innovation of measurements by allowing the exploration of other "unexpected" data.*

Response

The reviewer addresses highly relevant points and indeed we would see such an experienced programmer mentioned by the reviewer as part of the target audience of our toolbox. This is because the focus on modular architecture of the VNT enables programmers to easily take the parts they need, wherever they may profit from doing so and "start from scratch" where they do not. We have updated the introductory part of the manuscript to more clearly reflect these design goals and where the presented toolbox fits within the spectrum of different kinds of tools that can serve the research community (ll. 38-96). We have also added a new section describing the architecture of the VNT in more detail, to further illustrate the focus on ease of adaptation and how it facilitates code re-use in exactly the kind of scenario described by the reviewer (section "Designed to be changed: using code architecture to facilitate re-use" ll. 442-527).

1.4 Technical considerations: Unity versions

> *Since the tool needs to go through the Unity editor, this makes it sensitive to the version of the software used. I did not test it on all versions, but I already found incompatibility with common versions. This should probably be quickly discussed in the article.*

Response

The VNT makes use of modern features of the C# language and as such requires at least Unity version 2021.3 (LTS) to function. We have updated the manuscript to clarify this (l. 105).

1.5 Technical considerations: cross-platform deployment

> *The author say that the tool is "platform agnostic". I think this deserves more explanation. Is this the standalone build? Have the editor's tools been tested for windows mac and linux? The build for android and ios? Have this been tested on common VR systems like Oculus or HTC Vive ?*

Response

The tools of the VNT work independently of any 3rd-party components and can be easily adapted to be used in conjunction with driver software for

HMDs or other devices (see also response to next comment). As for creating standalone builds, the Unity engine allows compilation for most common target platforms (Linux, MacOS, Android, etc.) and to our knowledge no libraries used by the VNT preclude compilation for other platforms. However, the same is not true for the UXF package, which was used for the showcase experiment. This means that while the showcase can be compiled and run on other platforms, the UXF functionalities will not necessarily work on those platforms (we tested a build on Ubuntu 22.04.2 LTS and on macOS Monterey with M2 chip, where UXF file handling components did not function correctly). Thus, we have adapted the text to make it clear that the showcase study can only be expected to run on the Windows platform (ll. 708-712). However, we want to point out that the VNT tools can work independently of the UXF, so this limitation to the Windows platform is limited to the present showcase study, which makes use of the UXF.

1.6 Use of the term VR

> *I would not call the environment produced and used by the showcase study on a computer "immersive and flexible", and I would not even give it the name "virtual reality" Would these tools work in virtual reality for example via the use of the usual plug-ins like OpenVR SteamVR etc? VR is very promising in spatial cognition and I think any package oriented toward virtual navigation should also aim for virtual reality systems and not only personal computers. This deserves much more attention, I think, in the authors' article, as I would consider adding some discussion and references on why virtual reality is used in spatial cognition, including the ecological dimension it allows. For example (Cogné et al., 2017; Maneuvrier et al., 2020; Parsons, 2015)*

Response

We have updated the manuscript to make it more explicit, that our tools can be combined with different plug-ins like SteamVR etc. to target different presentation media (ll. 163-166, ll. 584-629). Indeed, we already use the VNT in conjunction with SteamVR and an HTC Vive Pro HMD. We created a version of the showcase experiment configured to use an HTC Vive Pro HMD that is available as a separate branch in the showcase repository and have stated as such within the manuscript (ll. 163-166). Due to the license of Unity Assets from the Asset Store we cannot provide a showcase version with

included SteamVR plugin, but instead provide instructions for integration in the repository's wiki.

Furthermore, we have expanded the discussion of different uses of VR and associated challenges (see also response to next comment) (ll. 584-629).

1.7 Remote deployment of experiments

> *Remote evaluation poses many methodological problems that are not mentioned at all in this article. This remote assessment also offers many promising avenues for diagnosis and rehabilitation, which are not mentioned any more than the problems. I think this deserves more attention as well, especially on the integration of tools such as VNT in this framework*

Response

We have expanded the discussion of remote deployment, its challenges and how they were met for the showcase study. We discuss these points in conjunction with the question of display media raised above (ll. 584-629).

1.8 Explanation of experimental tasks

> *In the showcase study, the triangle completion task is implemented and used but not really described. I would consider adding a word about the triangle completion task, its applications and uses and / or other virtual implementation of the task [Cherep et al., 2020, Dorado et al., 2019, McLaren et al., 2022, Riecke et al., 2002]. I would also consider describing in more details (with references) the traditional tasks covered by the tool.*

Response

We expanded the introduction and explanation of the triangle completion task accordingly (section "Triangle completion in virtual reality" ll. 129-147).

2 Review 2

> *The authors present an open source software toolbox to implement spatial navigation experiments using the Unity 3D engine. The*

manuscript first describes the requirements analysis, design decisions, and structure of the presented toolbox. The authors then present a validation experiment built using their toolbox and reimplementing a well-known spatial navigation task (triangle completion) with the additional component of a spatial pointing task. The manuscript is accompanied by two git repositories, one each for the toolbox and case study.

The software is novel, and the authors validate the tool on a well known task (fitting PLOS guidelines). The manuscript is clearly written and easy to follow, and the design decisions behind the toolbox become clear. I was especially happy to see that the authors focused on interoperability with existing tools (e.g., UXF), and that they include a full example study with additional documentation. However, some details about the case study were not fully clear to me in the current version, and I have included some other suggestions that might strengthen the manuscript below

2.1 desktop-VR vs. HMD

I realized only quite late in reading that the showcase study was desktop VR-based rather than run in an HMD (most recent studies seem to equate VR == headset). The authors might want to explicitly point this out earlier in the manuscript.

'*It would also be helpful to understand what parts of the toolbox apply to virtual environments in general (i.e., could also work in immersive VR), and which are desktop-specific*'

Response

We have updated the manuscript to make it more explicit that the showcase study was performed in desktop VR, but that the VNT is also compatible with HMDs and other presentation media (see also response to comment from reviewer 1 on same issue above) (ll. 163-166, ll. 584-629). Indeed, none of the features provided in the VNT (except for the mouse-based ray-casting, the replacement of which is discussed within the manuscript) (section "Example of interface driven design" ll. 500-527) are desktop specific and even the showcase study requires only minimal updating to work in HMD mode, (ll. 703-712) since the UI elements are designed in a manner enabling seamless switching of presentation media (world-space UI canvas "hovering" in front of camera, rather than screen-based UI).

2.2 Description of the showcase study

> *There are some details that would help the reader better understand the exact procedure of the showcase study (some of which might be in the wiki, but would be good to also detail in the manuscript): - How was the pointing task accomplished, i.e. how do participants "place a marker" (mouse cursor, aiming the viewport, ...)?*

Response

As part of the general restructure of the manuscript, we have added more details to the description of the showcase study procedure, both in the introduction to the task, (ll. 117-147) as well as as the trial walkthrough section (section "Showcase Part A: homing at different speeds" ll. 188-204, section "Novel Marker Placement task" ll. 175-187).

2.3 Results of the showcase study

'*The showcase Results section should state more clearly what statistical tests were performed for which experiment, and what the numerical results were*'

> *For example, the text mentions "Kruskal–Wallis one-way analysis of variance across all measures and conditions". Since velocity was the main IV, which of the pointing or endpoint errors was analyzed here? Alternatively, if this means a test was performed for each error metric, please be more explicit about this and provide the results for each test.*

'*The Fig. 4 caption test instead seems to suggest that error measures were compared within each avatar velocity? However, the numbers suggest that this is the same test as above. Please clarify.*'

'$p \leq 0.88126$*" might be a typo and indicates to p >=? In general, most statistical values seem to be written as "approximately equal", which seems unusual notation to me*'

'*The section describing participants and ethics approval on p. 9 does not mention any number of participants - please add.*'

Response

In complying with the suggestion of reviewer 1, we have removed all inferential statistics from the main manuscript (ll. 538-566) and made it more explicit that the showcase study is to be seen purely as a proof-of-concept for the toolbox (ll. 119-121, reduced detail in section "Results of the showcase study" (ll. 538-566), and reduced detail in section "Evolving the pointing task: Embedded 3D marker placement" (ll. 630-646)). However, a record of the statistical procedures originally performed is still available in the python scripts attached to the showcase repository (ll. 700-702).

2.4 Adding helpful information from the wiki to the main text

> *The case study wiki provides a lot of additional helpful information, some of which might be useful to include in the manuscript for readers still deciding whether this toolbox might work for their application. For example, a screenshot of the Unity scene hierarchy can give a person experienced with Unity a quick overview of the structure and objects/prefabs used.*

Response

We have considerably expanded the introduction to the toolbox overall, as suggested by reviewers, and have added a new figure showing the scene hierarchy of the showcase study (section "Technical Characterisation of the VNT" ll. 289-300, section "Designed to be changed" ll. 442-527, and Figure 4). However it is important to note that the modular nature of the VNT makes it possible to design different scene hierarchies for different projects and simply slot in VNT components wherever needed.

2.5 Discussion of other packages

'*In my opinion, the packages in Table 1 do not actually benefit from being presented as a table - I think the details in the table caption and references would combine to make a good descriptive paragraph in the manuscript.*'

Response

We have expanded the discussion of other packages and removed the table as suggested (ll. 38-96).

2.6 Creative commons licensing

'*I suggest naming the type of license explicitly in the text rather than just saying "open source" (I believe the abstract mentions it is CC)*'

Response

We have made it explicit, that the VNT is licensed under the GNU GPL 3 license (ll. 105-106)

2.7 Explanation of trap-lining

'*Since the authors mention a work-in-progress "trap-lining" study, it could be helpful to briefly mention what this means*'

Response

We have added a brief explanation, that trap lining refers to a type of route optimisation task, and given some references for the interested reader (Table 1 caption).

3 Review 3

In their manuscript, "The Virtual Navigation Toolbox: Providing tools for virtual navigation experiments," Martin M. Müller, Jonas Scherer, Patrick Unterbrink, Olivier J.N. Bertrand, Martin Egelhaaf, and Norbert Boeddeker present a new Unity 3D toolbox for designing spatial navigation experiments. The authors do a noteworthy job of putting together a new toolbox that is full of useful tasks, including a waypoint-based guidance task, a triangle completion task, and a novel pointing/marker placement task. Moreover, the authors describe data from two preliminary studies. First, they found a null difference in performance as a function of translation speed in an optic-flow-based triangle completion task. Second, they found that stable landmarks enhanced performance of the triangle completion task, thus replicating previous research. I commend the authors for their hard work on this project, and I have several questions and comments for the authors, which I hope will help strengthen the impact of their manuscript.

3.1 General structure

> *the manuscript felt to me like it was neither a comprehensive guide for their toolbox (i.e., the documentation herein was relatively sparse) nor did it feel like a full data paper (i.e., their sample sizes were very small: N=8 for Showcase A and N=10 for Showcase B)*
>
> *I felt that the overall scope of the discussion was a bit narrow, and I think the manuscript would have a stronger impact if the authors included more citations, background, rationale, etc. Therefore, I recommend that the authors consider either adding significantly more information about how to use their toolbox (i.e., a sort of guide introducing their software) and/or that they significantly increase the number of participants in their sample (i.e., a data paper in which they also introduce the overall functionality of the toolbox)*

'*As it stands, I personally feel that it is a bit too brief on both the background and the data side*'

Response

This recommendation matches those made by the other reviewers on the general structure of the manuscript. As previously stated, we have updated the whole manuscript to conform fully to the format of an introduction to the toolbox, rather than an empirical study (ll. 119-121, reduced detail in section "Evaluating of the showcase study" (ll. 530-566), and reduced detail in section "Evolving the pointing task: Embedded 3D marker placement" (ll. 630-646)). This includes expanding the description of tools and discussion of techniques, as suggested here (section "Designed to be changed: using code architecture to facilitate re-use" ll. 442-537).

3.2 Discussion of the "Landmarks" package

> *I personally felt that the authors' discussion of extant packages for spatial navigation was a bit misleading and inaccurate. For example, I will focus my discussion to Landmarks (i.e., their citation [30]) because I am very familiar with this package:*

a) *Lines 30-37: I disagree that users would have to "reinvent the wheel" in Landmarks. In fact, the key purpose of Landmarks is to allow users to generate commonly used spatial navigation experiments with VR (e.g., navigation tasks, several pointing tasks, map-drawing tasks). Also, yes, users can employ VR headsets but Landmarks makes it as simple as a click of a button in a dropdown menu to change between VR headsets and desktop versions of the task.*

b) *Lines 50-53: Again, I feel that Landmarks implements a very modular approach as well and all tasks/code/etc. can be recycled depending on the users' needs.*

c) *Lines 318-320: note that Landmarks can perform this functionality as well.*

d) *Lines 322-323: again, this sounds exactly like Landmarks.*

e) *Lines 325-328: This sounds like really great functionality (and, as far as I know, is not currently implemented in Landmarks).*

Response

We realise that the summary treatment of existing packages may have been misleading and therefore expanded both the discussion of existing packages, as well as the place of the VNT among them as part of the general restructure of the manuscript (ll. 38-96).

To briefly respond to the reviewer's comments on the Landmarks package specifically, we want to make it clear that we never intended to put into question the capabilities of that package. Our aim was to outline that the architectural differences between the VNT and other packages like Landmarks make it worthwhile to, among other things, provide functionalities which might already exist, because the VNT is designed to provide such functionalities in a way that makes them easier to adopt in new circumstances in isolation, without having to build one's project around the requirements of those parts of an existing package which one might not intend to use. This discussion of differing architectures and thus differing use cases is now the focus of the introduction of existing packages within the manuscript.

Concerning the implementation of HMD based VR experiments, we created a version of the showcase experiment configured to use an HTC Vive Pro HMD and SteamVR, that is available as a separate branch in the showcase repository and have stated as such within the manuscript (ll. 163-166). Due to the license of Unity Assets from the Asset Store we cannot include

the SteamVR plugin in our repository by including a simple button toggle between desktop and HMD usage. Instead, we provide instructions for integration of SteamVR in the repository's wiki.

3.3 Use of the term VR and modes of presentation

> *I feel that the authors use the term virtual reality (VR) a bit too broadly. For example, given the rise in highly immersive VR devices such as fully mobile VR headsets, I think it would help if the authors clarify that they are really talking about desktop-based navigation (i.e., to make it clear that this is a specific use case that does not include VR headsets).*
>
> *Relatedly, given the focus on desktop-based navigation, I think that it could severely limit the impact of their toolbox. Specifically, I apologize if I missed something, but it appears to me that the main task is a triangle completion task, which would allow researchers to study the role of optic flow, etc., on the path integration system. Therefore, I feel that the toolbox is limited in scope; e.g., given that other systems such as body-based cues should likely contribute to this process (e.g., as they cite in the papers from Harootonian et al.[5, 23]). However, I also understand that there were other aspects that users could employ for different types of tasks (e.g., the "waypoint-based guidance task" and the "pointing task" seemed like especially useful tasks).*
>
> *Related to my previous point, I might recommend that you are careful in referring to it as "path integration" because this appears to be a fully desktop-based task (i.e., only visual cues, no body-based cues). Do you envision that your toolbox will eventually be a fully VR-based task (i.e., with headset)? Either way, I recommend that you clarify these points.*

Response

As part of the general restructure, we have made it more explicit, that the VNT can be used with different peripherals and is indeed already in use with an HMD (see response to comment above (ll. 163-166). We also make it clear that we only refer to visual path integration (a term which is also used for example by [Wiener and Mallot, 2006]) in connection with the showcase study, as no proprioceptive cues were available in desktop-VR (ll.

197-199). Finally, we have added a discussion of different modes of VR to better place the showcase study in the context of existing work (section "Performing distributed virtual navigation experiments", ll. 584-629).

3.4 Implementation of new tasks

'*Lines 229-230 and 305: The authors discuss the idea that users could make new tasks and functionality with "minimal integration work." How much work? Also, what would be the nature of such work (e.g., coding in C#)?*'

Response

As part of the general restructure, we added a new section discussing the technical aspects of working with the toolbox, including an example of the workflow of integrating new functionalities into the VNT (section "Designed to be changed: using code architecture to facilitate re-use" l.442-527 and "Example of interface driven design" ll. 500-527 in particular).

3.5 Package Maintenance

> *Similarly, given that maintaining software packages requires a lot of effort, resources, and personnel, what plans/frameworks do you have in place to ensure the longevity of this package? (I see that you discuss that the general community can contribute, but how likely do you think this would be to occur?)*

Response

Maintaining software packages is indeed a universal challenge. We have taken several steps to make the future maintenance of the VNT as easy as possible. Firstly, we are deeply convinced that good software development is collaborative rather than competitive. For this reason we have made the VNT available as an open source package on gitlab (section "Data Availability" ll. 686-712). Providing tools freely is the prerequisite of achieving any community involvement. Secondly, to make it easier for members of the public to contribute to the VNT, the available modules are extensively documented, including class graphs visualising the code structure of each module and demos to see how the different components work together in practise. Finally, the VNT forms the basis of the VR work going on within the associated labs at Bielefeld university. As such, we feel confident that the VNT will

be maintained and expanded even without external community engagement, since it is in daily use.

3.6 Marker placement task

> *I feel that the authors are selling themselves short by referring to their task as a "pointing task". Generally, we tend to refer to pointing tasks as those in which participants point in a given direction. Instead, I might recommend that the authors refer to this as a "marker placement task" or something of that nature to make it clearer that this is a novel task and that it can offer additional information (i.e., about direction and distance simultaneously). Also, I felt that the authors could add a more complete description of their task.*

Response

We updated and expanded the description of the task and adopted the term suggested by the reviewer (section "Novel marker placement task" ll. 175-187).

3.7 Description and Analysis of the showcase task

> *In general, I felt that the Methods section could be more detailed to explain both the tasks and the participants. For example, will you please clarify the following points?*
>
> *a) The authors only mention the sample sizes in the figure captions. Moreover, why did the number of participants differ between Showcase A and B (were these separate groups of participants)?*
>
> *a) Also, I was unclear on the description of the number of trials in the task (i.e., based on the methods). At first, it sounded like there were only two trials, but then the figure captions (e.g., for Figure 4 indicates 17-24 repetitions) and results listed a different number of trials. How many trials did the participants complete (and why were there differing trial counts between participants)?"*
>
> *As far as the data in this paper go, I again feel that the sample size is quite small (N=8 for Showcase A and N=10 for Showcase B), which I think is a limitation. For example:*

a) *Lines 364-369: the null result here might not justify combining results due to the extremely small sample size and extremely small trial count. Also, the* $p > 0.1$ *might actually be trending toward a difference? Were the trial orders counterbalanced? Was there any sign of an effect in which participants improved on the second trial (or subsequent trials; again, sorry but I am unclear on the trial structure here [e.g., what do you mean by, "or in a design which mirrored the spatial layout along the world y-axis, yielding trials with either left or right-handed turns, which were presented in randomised order," in the caption of Figure 1]?*

a) *For the null result of translation velocity: i. Should you consider a Bayes null analysis here (i.e., to determine whether the evidence is in favor of the null)? ii. Again, the small sample size is a concern for me, especially when you are interpreting null effects (i.e., could this simply be a power issue?). Therefore, at a minimum, I would recommend tempering your conclusions here (but as I mention above, I recommend adding more participants to your sample).*

Response

We restructured the manuscript to make it more explicit that the showcase study is to be seen as a prove-of-concept rather than a full empirical study (ll. 119-121, reduced detail in section "Evaluating of the showcase study" (ll. 528-566), and reduced detail in section "Evolving the pointing task: Embedded 3D marker placement" (ll. 630-646)). Furthermore, we have expanded the description of the showcase to better summarise the procedure (ll. 188-211, section "Novel Marker Placement task" ll. 175-187, section "Triangle completion in virtual reality" ll. 129-147).

To briefly answer the question of the reviewer regarding the structure of the task:

- The full experimental protocol consisted of 4 sessions (preceded by 1 training session), each containing 6 (3 left, 3 right) repetitions of each experimental condition (trans. vel.or num. obj. respectively). Thus, a full data set for one participant contains 24 trials per condition.
- The actual number of repetitions differs from 24 for some participants, because not all of them fully completed all sessions of the experiment. However, since conditions were intermixed throughout, we were able to

evaluate the results even if single sessions were missing for individual participants.

3.8 Comprehensiveness of the discussion

'*For the landmark part of discussion, etc., I feel that you could cite more papers. Again, I feel that you could generally supplement your manuscript throughout to make it more comprehensive (including more citations).* '

Response

As part of the general restructure of the manuscript, we have expanded the discussion and contextualisation of the toolbox and showcase study overall (e.g. ll. 38 - 96 for a detailed discussion on existing packages and embedding of the VNT therein, or section "Performing distributed virtual navigation experiments" ll. 584-629).

3.9 Minor points

'*Lines 206-208: I was initially a bit unclear about the nature of the task. Here, I surmised that they performed the "pointing task" twice, but the description here was a bit unclear for me.*'

'*Line 210: what were the parameters for the "timeout" state?*'

'*Line 354: quotation ends funny (i.e., should be right after "Data Availability" to close quotes).*'

Response

The issues were addressed as part of the general restructure of the manuscript. To briefly answer both of the questions here as well:

- The pointing task was indeed performed twice per trial.Once at the start of the return and then once again after the participant had moved more than 12.5 meters away from the start of the return (12.5 meters were half of the straight-line distance from the start of the return to the goal) (ll. 192-195).
- The timeout state was triggered if the participant took more than 60 seconds to lock in a goal estimate during the "homing" phase of the trial.This timer was paused during the embedded pointing task (ll. 285-286).

References

[Amrhein et al., 2019] Amrhein, V., Greenland, S., and McShane, B. (2019). Scientists rise up against statistical significance. *Nature*, 567(7748):305–307.

[Cherep et al., 2020] Cherep, L. A., Lim, A. F., Kelly, J. W., Acharya, D., Velasco, A., Bustamante, E., Ostrander, A. G., and Gilbert, S. B. (2020). Spatial cognitive implications of teleporting through virtual environments. *Journal of Experimental Psychology: Applied*, 26(3):480.

[Colquhoun, 2017] Colquhoun, D. (2017). The reproducibility of research and the misinterpretation of p-values. *Royal society open science*, 4(12):171085.

[Dorado et al., 2019] Dorado, J., Figueroa, P., Chardonnet, J.-R., Merienne, F., and Hernández, T. (2019). Homing by triangle completion in consumer-oriented virtual reality environments. In *2019 IEEE Conference on Virtual Reality and 3D User Interfaces (VR)*, pages 1652–1657. IEEE.

[McLaren et al., 2022] McLaren, R., Chaudhary, S., Rashid, U., Ravindran, S., and Taylor, D. (2022). Reliability of the triangle completion test in the real-world and in virtual reality. *Frontiers in Human Neuroscience*, 16:945953.

[Riecke et al., 2002] Riecke, B. E., Veen, H. A. v., and Bülthoff, H. H. (2002). Visual homing is possible without landmarks: A path integration study in virtual reality. *Presence: Teleoperators & virtual environments*, 11(5):443–473.

[Wiener and Mallot, 2006] Wiener, J. M. and Mallot, H. A. (2006). Path complexity does not impair visual path integration. *Spatial cognition and computation*, 6(4):333–346.
